# Evaluation of *Euglena gracilis* 815 as a New Candidate for Biodiesel Production

**DOI:** 10.3389/fbioe.2022.827513

**Published:** 2022-03-25

**Authors:** Zixi Chen, Yehua Chen, Hua Zhang, Huan Qin, Jiayi He, Zezhou Zheng, Liqing Zhao, Anping Lei, Jiangxin Wang

**Affiliations:** ^1^ Shenzhen Key Laboratory of Marine Bioresources and Eco-environmental Science, Shenzhen Engineering Laboratory for Marine Algal Biotechnology, Guangdong Provincial Key Laboratory for Plant Epigenetics, College of Life Sciences and Oceanography, Shenzhen University, Shenzhen, China; ^2^ Shenzhen Academy of Environmental Science, Shenzhen, China; ^3^ College of Chemistry and Environmental Engineering, Shenzhen University, Shenzhen, China

**Keywords:** microalgae, *Euglena gracilis* 815 strain, paramylon, fatty acids, biodiesel

## Abstract

*Euglena* comprises over 200 species, of which *Euglena gracilis* is a model organism with a relatively high fatty acid content, making it an excellent potential source of biodiesel. This study isolated and characterized a new strain named *E. gracilis* 815. *E. gracilis* 815 cells were cultivated under light and dark conditions, with either ethanol or glucose as an external carbon source and an autotrophic medium as control. To achieve maximum active substances within a short period i.e., 6 days, the effects of the light condition and carbon source on the accumulation of bioactive ingredients of *E. gracilis* 815 were explored, especially fatty acids. In comparison with the industrially used *E. gracilis* Z strain, *E. gracilis* 815 exhibited high adaptability to different carbon sources and light conditions, with a comparable biomass and lipid yield. The content and composition of fatty acids of *E. gracilis* 815 were further determined to assess its potential for biodiesel use. Results suggested that *E. gracilis* 815 has biodiesel potential under glucose addition in dark culture conditions and could be a promising source for producing unsaturated fatty acids. Therefore, *E. gracilis* 815 is a candidate for short-chain jet fuel, with prospects for a wide variety of applications.

## Introduction

Extensive global industrialization has driven an increasing energy demand and caused a severe energy crisis. Therefore, alternative and renewable sources of energy are urgently needed. Estimates suggest they can provide 30% of the worldwide energy demand without compromising on food production (Koonin, 2006). Since biomass energy is massive, renewable, and environmentally friendly (Wu et al., 2012; Chew et al., 2017; Chandra et al., 2019; Dębowski et al., 2020a), it has become an essential source of alternative energy (Williams, 2007; Li et al., 2008).

Biodiesels are complex in composition and mainly include palmitic acid, stearic acid, oleic acid, linoleic acid, and other long-chain fatty acids and esters formed by alcohol (Vicente et al., 2007). Rising demand for traditional biofuels, which were originated from food crops such as soybeans, corn, rapeseed, and castor oil, has inadvertently worsened the food crisis. Microalgae biodiesel may be a solution to this problem (Brennan and Owende, 2010; Lei et al., 2012). Microalgae convert light energy into chemical energy, subsequently storing energy as lipids in their cells (Chisti, 2007; Lam and Lee, 2012; Kang et al., 2022; Li et al., 2022). Since the carbon chain lengths of these lipids are similar to that of diesel, these lipids can be transformed into biodiesel via transesterification (Chisti, 2007; Ramos et al., 2009; Jung et al., 2021; de Carvalho Silvello et al., 2022). Because fatty acids are the precursors for biodiesel production, many researchers in this area have focused on comparing the content and composition of fatty acids among different oleaginous algae (Harwood and Guschina, 2009). Other studies have shown that fatty acid composition significantly impacts the fuel characteristics of biodiesel (Ramos et al., 2009), including essential indicators for evaluating biodiesel potentials such as cetane number (CN), iodine value (IN), and saponification value (SN) (Lei et al., 2012).

*Euglena* species lack cell walls and have high nutrient availability (Zakryś et al., 2017; Shao et al., 2019). They could be cultivated on a large scale and used in various industrial applications (Mahapatra et al., 2013; Sun et al., 2018; Wu et al., 2020; Wu et al., 2021b). Some *Euglena* species produce medium and long-chain fatty acids, making them potential candidates for having biodiesels to meet the demands of the energy market, and such as in jet fuel (Zeng et al., 2016; Gupta et al., 2021).

*Euglena* species are highly adaptable and evolutionarily distinct. Notably, *Euglena* species possess characteristics of both plants and animals, and they can grow autotrophically, mixotrophically or heterotrophically with glucose, ethanol, glutamic acid, malate, pyruvate, lactate, and other carbon sources (Barsanti et al., 2000). Some studies on *Euglena* have shown that the addition of a carbon source can induce cell division and promote growth (Hurlbert and Rittenberg, 1962; Rodríguez-Zavala et al., 2006; Wu et al., 2021b), as well as increase the content of total lipids, mainly neutral lipids (Coleman et al., 1988; Thuillier-Bruston et al., 1990). Mainly, organic carbon sources such as ethanol and glucose have been found to participate in different metabolic pathways of *Euglena* and strongly influence their growth and accumulation of active substances. When *Euglena* metabolizes ethanol, it is rapidly oxidized to acetate and converted into acetyl-CoA and participates in the tricarboxylic acid cycle (TCA cycle) (Garlaschi et al., 1974; Zimorski et al., 2017). In comparison, glucose is mainly metabolized through glycolysis and pentose phosphate pathways (PPP) similar to that of other organisms (Hurlbert and Rittenberg, 1962).

The industrial use of *Euglena* has developed rapidly in recent years owing to its commercial potential and value. However, the selection and culture of different *Euglena* species remain a significant obstacle, and most strains have limited application capabilities. Therefore, efforts to screen and identify excellent *Euglena* strains are crucial to expand the range of potential industrial applications for *Euglena* and reduce production costs (Suzuki et al., 2015).

In this study, a new *E. gracilis* strain, *E. gracilis* 815, which uses glucose and ethanol as additional carbon sources, was isolated and cultured under both dark and light conditions. To achieve a high yield of active substances within a short period (i.e., a 6-day culture period), the effects of nutrient and light conditions on the growth, biomass, paramylon, and total lipid accumulation of *E. gracilis* 815 were investigated. Meanwhile its fatty acid composition and contents were also determined. To assess the potential value of *E. gracilis* 815 for biodiesel applications, its properties were compared with that of the industrially used *E. gracilis* Z strain. Finally, the potential for the industrial application of *E. gracilis* 815 was discussed and evaluated.

## Materials and Methods

### Isolation and Cultivation of *Euglena* Strains

The *Euglena* strain was collected from water samples taken from a fishpond in Fuzhou, China (26°08′N, 119°28′E) in August 2018. Single cells were separated using a capillary pipette and cultured in 96-well plates. They were cultivated autotrophically using Cramer-Myers (CM) medium (Cramer and Myers, 1952) under 25°C and 80 μmol photons·m^−2^·s^−1^.

### Culture Medium and Growth Conditions

The culture of *E. gracilis* 815 strain at log phase were inoculated into a 1 L Erlenmeyer flasks containing 250 ml fresh CM medium. Two carbon sources which were commonly used in the cultivation of *E. gracilis* Z (Rodríguez-Zavala et al., 2006; Rodriguez-Zavala et al., 2010), glucose (15 g/L) or ethanol (1% v/v), were added after the medium was autoclaved and cooled for the static culture of *E. gracilis* 815. The autotrophic group was cultured under autotrophic light conditions (CM(L), 80 μmol photons·m^−2^·s^−1^), while the mixotrophic and heterotrophic groups were cultured as follows: ethanol + light (CM + E(L), 1% v/v, 80 μmol photons·m^−2^·s^−1^), glucose + light (CM + G(L), 15 g/L, 80 μmol photons·m^−2^·s^−1^), ethanol + darkness (CM + E(D), 1% v/v, 0 μmol photons·m^−2^·s^−1^), and glucose + darkness (CM + G(D), 15 g/L, 0 μmol photons·m^−2^·s^−1^). Apart from the parameters described above, all groups were statically cultured under 25°C.The initial density in each group was set to 1.7 × 10^5^ cells/ml. Three replicates of each group were cultured simultaneously.

To further compare the biomass accumulation between *E. gracilis* 815 and *E. gracilis* Z, these two strains were cultivated in 1.2 L photobioreactors containing 600 ml fresh CM medium and 1% v/v ethanol as carbon sources. All samples were grown under 25°C, with 80 μmol photons·m^−2^·s^−1^ white light and bubbled with 12 L/min air, while the initial density was 1.1 × 10^6^ cells/mL. After 6 days, cells were collected, dried, and weighted to compare the biomass.

### Cell Growth Monitoring

From Day 0 to the day when all groups came to the plateau phase, samples were collected daily. Before sampling, the algae culture in the Erlenmeyer flask was shaken gently. Then, 1 ml of the algae liquid was transferred into a 1.5-ml EP microtube, and counted using a phase-contrast inverted microscope.

### Measurement of Biomass

The photosynthetic autotrophic group reached the plateau phase on Day 6 of cultivation. To ensure data integrity, physiological and biochemical analyses of *E. gracilis* 815 cells were only carried out on Day 6. Totally 50 ml of *E. gracilis* 815 or *E. gracilis* Z cells were transferred from each flask to a pre-weighted 50-ml centrifuge tube. Algal cells were centrifuged at 8,000 rpm for 3 min, collected, lyophilized, and weighted for biomass.

### Determination of Paramylon Content

20 mg of *E. gracilis* 815 lyophilized algae powder was transferred into a 15-ml glass centrifuge tube, mixed with 4 ml acetone on a vortex mixer twice (15 s each time), shook at 150 rpm for 1 h, and centrifuged at 5,000 rpm for 5 min. After aspirating the supernatant, the pellet was resuspended with 1 ml of 1% SDS solution, transferred to a pre-weighed 1.5-ml EP tube, and kept at 85°C for 30 min. Then the sample was centrifuged at 2000 rpm for 5 min. After discarding the supernatant, the sample was placed in an oven at 50°C for drying to a constant weight, and the dried powder was paramylon (Takenaka et al., 1997; Wu et al., 2020; Wu et al., 2021b).

### Total Lipids Determination

The modified Bligh-Dyer method extracted and determined the total lipids (Bligh and Dyer, 1959). Totally 20 mg of *E. gracilis* 815 dry algae powder was transferred into a 15-ml centrifuge tube. To lyse the algal cells and extract the lipids, 9.5 ml of mixed solvent (chloroform: methanol: distilled water = 1: 2: 0.8) was added to the powder. Then the sample was shaken vigorously for 5 min, ultrasonicated for 30 min, and centrifuged at 5,000 rpm for 2 min. After collecting the supernatant, the remaining algae cells were precipitated, followed by repeating the extraction step twice. Then distilled water and chloroform were added to the collected extract and mixed to achieve a final ratio of chloroform: methanol: distilled water as 1:1:0.9. After mixing, the sample was centrifuged at 5,000 rpm for 10 min. The lower chloroform layer containing lipids was collected to a pre-weighed 50-ml glass tube and dried to constant weight by nitrogen blowing. The total lipid yield was then calculated based on the biomass.

### Fatty Acid Composition Analysis

Totally 10 mg of *E. gracilis* 815 dry algae powder was used to quantitatively analyze fatty acid composition. To lyse the algae cells, algae powder was transferred into a glass tube, followed by adding 1 ml of 2 mol/L NaOH-CH_3_OH solutions and 50 μl of methyl nonadecanoate working solution (5 mg of methyl nonadecanoate dissolved in 10 ml of dichloromethane) as the internal standard, vortexing for 30 s on a vortex shaker, and shaking for a further 1 h on a shaker at 110 rpm. Cell lysate was placed in a 75°C water bath for 30 min for saponification, and cooled down naturally at room temperature. For methyl esterification, 1 ml of 4 mol/L HCl-CH_3_OH solution and 0.5 ml of concentrated HCl (mass fraction of 38%) were added to the saponified lysate to achieve the sample pH < 2, then the sample was kept in a water bath at 75°C for 30 min, and cooled down naturally at room temperature. Then, 1 ml of n-hexane was added for extracting the lipids, followed by vortexing the sample for 5 min. The sample was centrifuged at 4,000 rpm for 2 min, and the supernatant was transferred to a new glass tube, followed by repeating the extraction step twice. The supernatants were combined, filtered through a 0.22 μm PVDF filter into a new test tube, and the solvent was blown dry with nitrogen. A total of 500 μl of dichloromethane was added into the test tube to fully dissolve the fatty acid methyl ester. The dissolved sample was quickly transferred to the chromatography injection bottle for GC-MS analysis.

A gas chromatography-mass spectrometer (GC-MS, Agilent 7890A-5975C) detected the fatty acid composition. The chromatographic column VF-23 ms (30 m × 320 μm × 0.25 μm) was used, and high-purity helium gas with the purity greater than 99.999% was used as the carrier gas. The injection volume was 2 μl, with a 3 min solvent delay. The injection port temperature was 240°C, the injection port pressure was 1.2 psi, the column flow rate was 1.2299 ml/min, and the split ratio was 10:1. The heating program was as follows: the initial temperature was set at 70°C and held for 1 min, increased to 180°C at a rate of 25°C/min and held for 2 min, increased to 205°C at a rate of 3°C/min and held for 2 min, then increased to 230°C at a rate of 8°C/min and kept for 5 min (Wang et al., 2013; Zeng et al., 2016).

Before loading each batch of samples, 37 kinds of fatty acid mixed standards (Sigma, catalog number: CRM47885) were tested. The chromatographic peaks of the mixed standard samples were qualitatively and quantitatively analyzed in combination with Agilent ChemStation software (Shao et al., 2019).

### Calculation of CN, IN, and SN Values

The SN, IN, and CN values were calculated according to the fatty acid composition and content on Day 6 to evaluate the biodiesel potential of *E. gracilis* 815 cultured under different conditions. The SN, IN, and CN values were calculated using the empirical using the empirical formulae (1–3) (Krisnangkura, 1986; Azam et al., 2005).

SN=∑(560×Pi)/MWi (1)

IN=∑(254×D×Pi)/MWi (2)

CN=46.3+5458/SN−0.225×IN (3)

Pi refers to the weight percentage of each fatty acid, MWi refers to the molecular weight of each fatty acid, and D refers to the number of double bonds in each fatty acid (Lei et al., 2012).

### Statistical Analysis

The mean value and standard deviation (SD) across the three replicates in each group were calculated. One-way analysis of variance (ANOVA) was used to test the significance of differences in cell density, cell biomass, paramylon and total lipids, fatty acid composition, SN, IN, and CN under different conditions. When the variances were homogeneous, and the differences among other states were significant (*p* ≤ 0.05), the Student-Newman-Keuls multiple comparison tests were used to determine the source of the differences. When the variances were not homogeneous, the Dunnett’s C test was used for analysis and comparison. SPSS 17.0 software (SPSS Inc., United States) was used for all the statistical analyses.

## Results

### Species Identification and Morphological Observation

**FIGURE 1:**
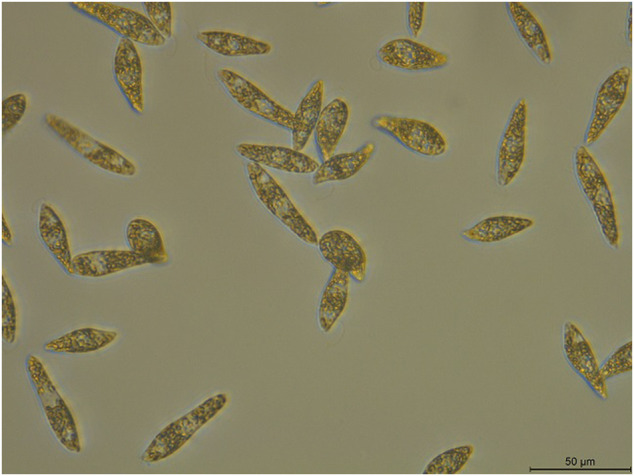
Morphology of *E. gracilis* 815. Scale bar: 50 μm.

To identify the newly isolated algal strain, the 18S rRNA sequence of the samples were sequenced. According to the 18S rRNA sequencing results (GenBank sequence numbers LSU: MW690035; SSU: MW690034), the samples were identified as *E. gracilis*, and this strain was designated as *E. gracilis* 815. Individual *E. gracilis* 815 algal cells, with approximately 30–50 μm in length and 10–20 μm in width, were slender and spindle-shaped with tapered ends, lacked cell walls (Figure 1), and could swim freely.

### Growth and Biomass of *E. gracilis* 815 Under Different Conditions

**FIGURE 2:**
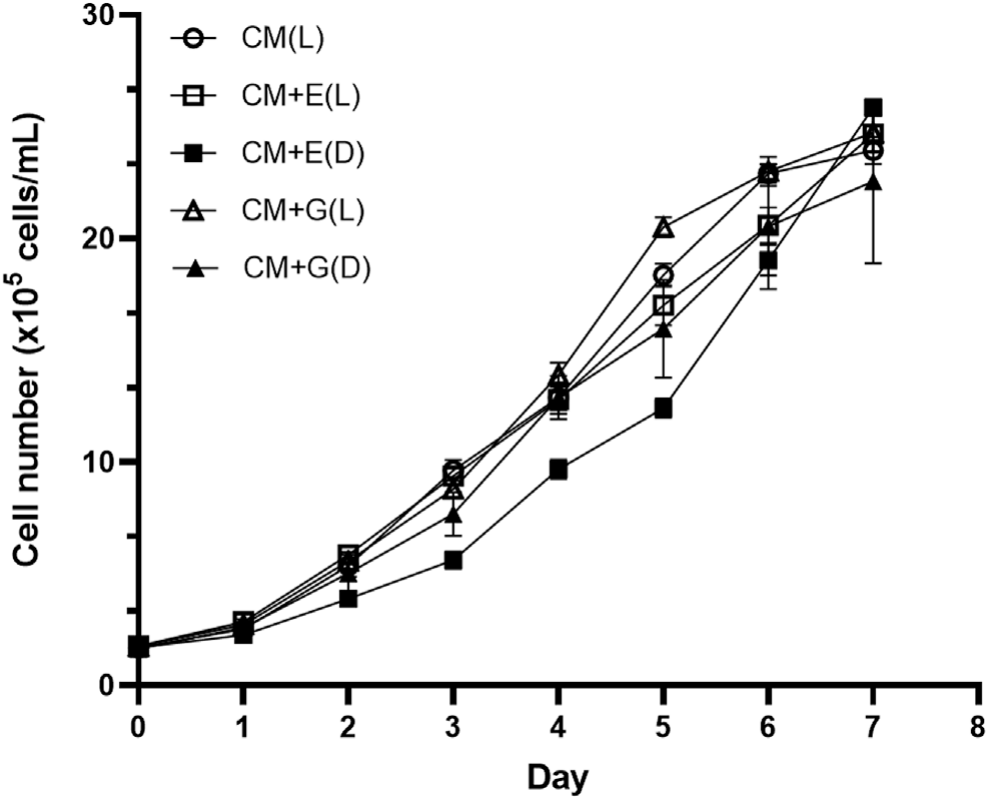
Growth of *E. gracilis* 815 under different conditions. Abbreviations: CM(L), CM medium + light; CM + E(L), CM medium + ethanol (light); CM + E(D), CM medium + ethanol (dark); CM + G(L), CM medium + glucose (light); CM + G(D), CM medium + glucose (dark). Values correspond to the mean ± standard deviation (n = 3).

**FIGURE 3:**
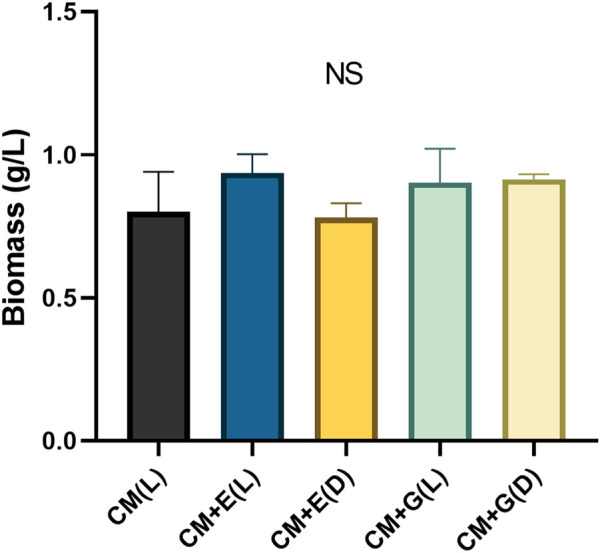
Biomass (g/L) of *E. gracilis* 815 cultured under different conditions on Day 6. NS indicates no significant difference according to ANOVA test at *p* > 0.05.

To evaluate the ability of using different carbon sources under light or dark conditions, *E. gracilis* 815 was cultured under autotrophic (CM(L)), mixotrophic (CM + E(L) and CM + G(L)), and heterotrophic (CM + E(D) and CM + G(D)) conditions. Growth of the autotrophic groups reached the plateau phase on Day 6, when cell densities ranged from 1.90 × 10^6^ cells/ml (CM + E(D)) to 2.30 × 10^6^ cells/mL (CM + G(L)). The CM + E(D) group grew slowly between Day2 to Day5, but there were no significant differences among different conditions on Day 6 (Figure 2). In addition, no significant differences were observed among different conditions in the accumulation of cell biomass, which ranged between 0.78–0.94 g/L on Day 6 of incubation (Figure 3).

### Paramylon Quantification

**FIGURE 4:**
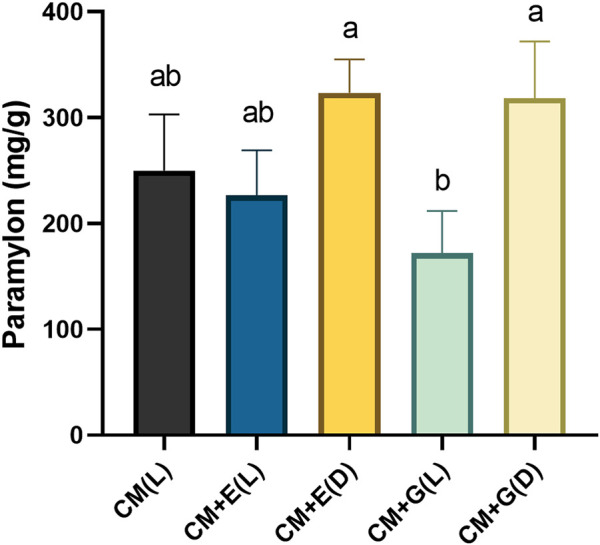
Paramylon content (mg/g dw) of *E. gracilis* 815 cultured under different conditions on Day 6. Different letters indicate significant difference according to ANOVA test at *p* ≤ 0.05.

*E. gracilis* 815 cells were collected on Day 6, and lyophilized algae powder was used for extracting paramylon according to the methods described above. Heterotrophic groups, using ethanol or glucose as carbon sources and cultivated in the dark, significantly accumulated more paramylon than the mixotrophic group using glucose and cultivated in light (*p* ≤ 0.05) (Figure 4). However, no significant differences were observed among the three autotrophic and mixotrophic groups cultivated in light. Quantitatively, the paramylon content of the two heterotrophic groups ranged between 318.3–323.3 mg/g dw (dried weight), and the three autotrophic and mixotrophic groups yielded paramylon between 171.7–250 mg/g dw (Figure 4). In summary, heterotrophic cultivation should be used to produce paramylon in *E. gracilis* 815, which agreed to the previous results in *E. gracilis* Z (Zeng et al., 2016).

### Total Lipids Quantification, Fatty Acid Content and Composition

**FIGURE 5:**
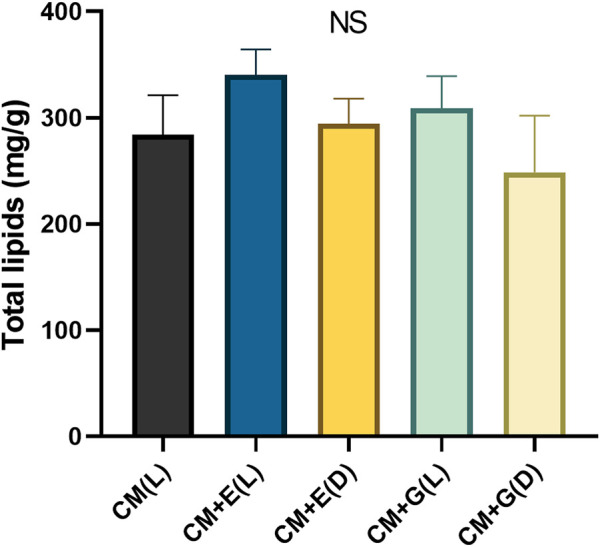
Total lipid contents (mg/g dw) of *E. gracilis* 815 cultured under different conditions on Day 6. NS indicates no significant difference according to ANOVA test at *p* > 0.05.

**FIGURE 6:**
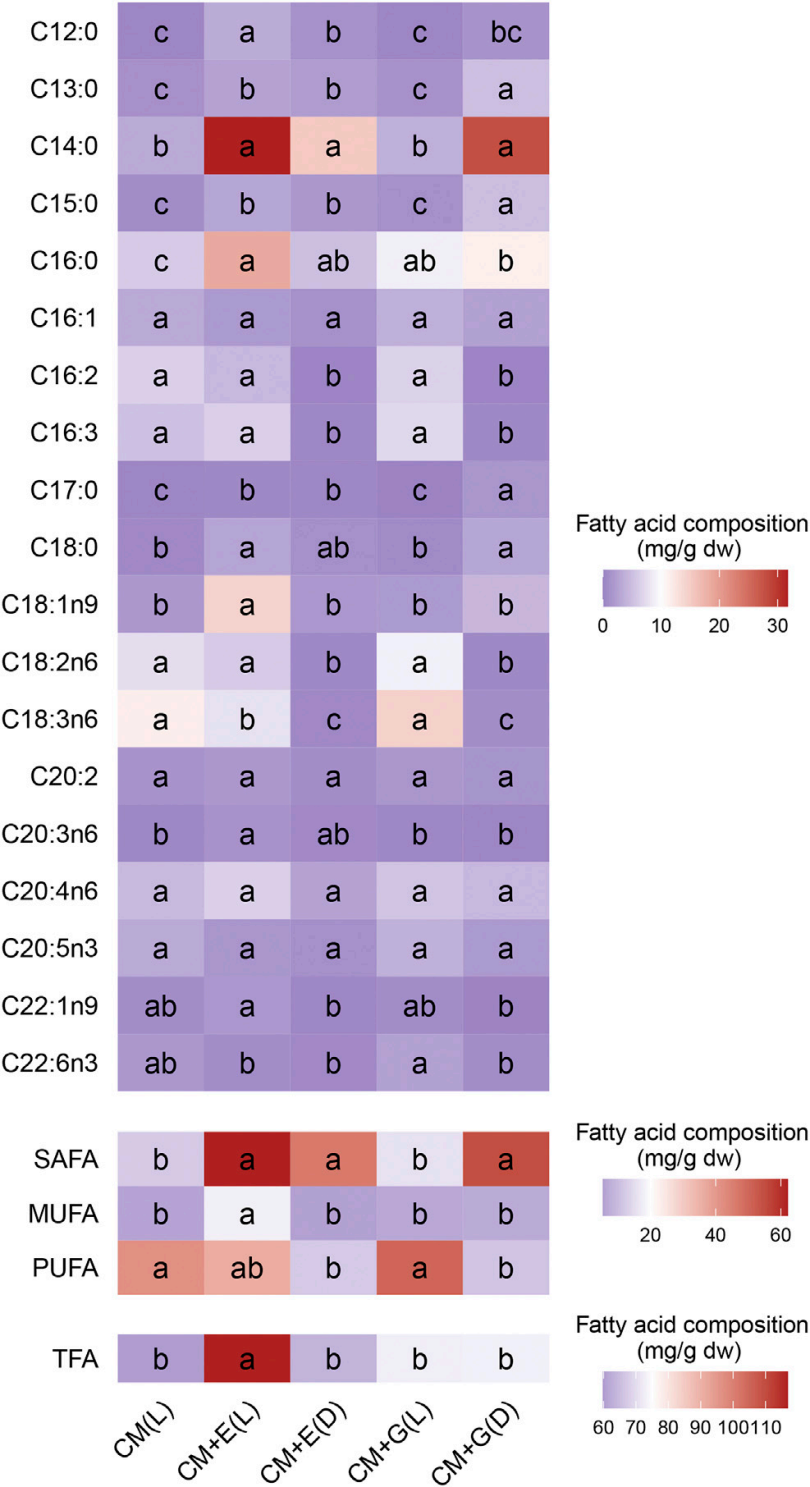
Heatmap of fatty acid composition (mg/g dw) of *E. gracilis* 815 cultured to Day 6 under different conditions. Different letters indicate significant difference according to ANOVA test at *p* ≤ 0.05.

The five groups yielded total lipids between 248.67–340.33 mg/g dw on Day 6 (24.87–34.03% total lipids in dry weight), but no significant differences were observed. However, yields from the two mixotrophic groups (340.33 mg/g dw for CM + E(L) and 309 mg/g dw for CM + G(L)) were slightly higher than those from the autotrophic group (284 mg/g dw for CM(L)) and two heterotrophic groups (294.33 mg/g dw for CM + E(D) and 248.67 mg/g dw for CM + G(D)) (Figure 5). To further evaluate the potential of producing fatty acids using *E. gracilis* 815, the fatty acid contents and compositions cultivated under different conditions according to the methods described above (Figure 6 and Supplementary Table S1). Totally 19 individual fatty acids were detected, and the fatty acid carbon chain composition was C12-C22. Although only the mixotrophic group with ethanol exhibited significantly higher total fatty acid (TFA) content (*p* ≤ 0.05), there were significant differences in the contents of different types of fatty acids among all groups. Compared with the autotrophic group, cells cultivated with ethanol significantly accumulated more saturated fatty acids (SAFAs) (*p* ≤ 0.05), which accounted for 50–70% of total fatty acid content. In addition, the content of monounsaturated fatty acids (MUFAs) in the mixotrophic group using ethanol were significantly higher (*p* ≤ 0.05) than the others. In groups using glucose, polyunsaturated fatty acids (PUFAs) were mainly found to accumulate in the presence of light, while SAFAs accumulated primarily in the dark. The autotrophic and the mixotrophic groups using glucose accumulated significantly higher (*p* ≤ 0.05) levels of PUFAs, which accounted up to 70% of total fatty acid content, and these two conditions could be the candidates for the further study evaluation of producing PUFAs.

When *E. gracilis* 815 were cultured in light, significant differences in fatty acid content were observed between the autotrophic and mixotrophic groups. Compared with the autotrophic group, most types of fatty acids in the mixotrophic group using ethanol were significantly higher (*p* ≤ 0.05). These included medium and long-chain SAFAs (C12-C18), as well as unsaturated fatty acids (UFAs), such as oleic acid (C18:1n9), and C20:3n6. However, the contents of most types of fatty acids in the mixotrophic group using glucose did not differ significantly from those of the autotrophic group.

When *E. gracilis* 815 was cultured in the dark, the heterotrophic group using ethanol significantly accumulated fewer fatty acids than the mixotrophic groups cultured in light (*p* ≤ 0.05). Most of these differences were attributed to a decrease in the contents of UFAs, such as C16:2, C16:3, oleic acid (C18:1n9), linoleic acid (C18:2n6), α-linolenic acid (C18:3n6), erucic acid (C22:1n9), and others. Similarly, the heterotrophic group using glucose also significantly accumulated less UFAs than the mixotrophic group, such as C16:2, C16:3, linoleic acid (C18:2n6), α-linolenic acid (C18:3n6), and DHA (C22:6n3). In addition, the two heterotrophic groups accumulated significantly higher (*p* ≤ 0.05) levels of medium and long-chain SAFAs such as C13-C18.

### Evaluation for the Biodiesel Potential of *E. gracilis* 815

As *E. gracilis* 815 accumulated various kinds of fatty acids under different conditions, several critical properties of biodiesels, including SN, IN, and CN values were assessed. SN refers to the mass of potassium hydroxide required for the complete saponification of one unit of oil, IN refers to the number of grams of iodine absorbed per 100 g of oil (reflecting the degree of unsaturation of the oil), and CN is an essential indicator for evaluating the ignition performance of diesel. SN and IN can be used to characterize the possibility of fatty acids or fatty acid methyl esters as raw materials for biodiesel and are related to the length of the fatty acid carbon chain and the degree of unsaturation. CN, the leading indicator of biodiesel quality, can be used to evaluate the impact of biodiesel on the stability and combustion process of diesel engines (Lu et al., 2012).

**FIGURE 7:**
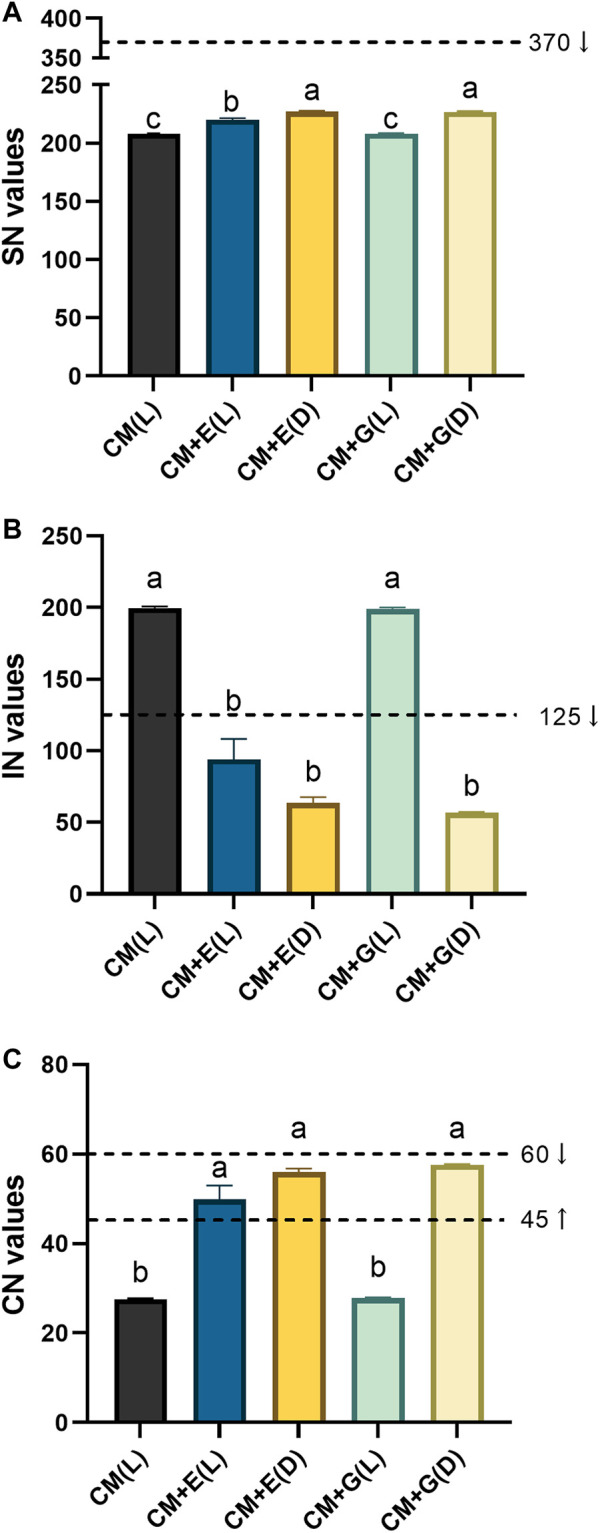
Quality evaluation of biodiesel from *E. gracilis* 815 cultured under different conditions on Day 6. **(A)** SN values; **(B)** IN values; **(C)** CN values. Different letters indicate significant difference according to ANOVA test at *p* ≤ 0.05. Arrows indicate the required ranges of these values in several standards.

The SN values of each group of *E. gracilis* 815 ranged between 208.1–227.1 (Figure 7A). The current American ASTM D6751 standard requires an SN standard of biodiesel to be less than 370 (Sakthivel et al., 2018). While there were significant differences between different groups, all groups met this requirement. The IN values of mixotrophic and heterotrophic groups varied substantially, ranging between 56.6–200 (Figure 7B). National biodiesel standards require an IN value not exceeding 125 (Luo et al., 2006). The heterotrophic groups and the mixotrophic group using ethanol, met this requirement. The CN values of each group ranged between 27.6–57.6 (Figure 7C). The international minimum standard for biodiesel CN is set at a value of 45, and values between 45–60 are considered suitable. Similar to IN, the heterotrophic groups and the mixotrophic group using ethanol met this requirement. Based on these results, the heterotrophic cultivation and the mixotrophic cultivation using ethanol could be the candidates for the further evaluation of producing biodiesel.

## Discussion

Carbon sources and photosynthesis influence the growth of *Euglena*. No significant differences in cell density between groups of *E. gracilis* 815 using photosynthetic autotrophy, as well as groups using ethanol or glucose as carbon sources were observed on Day 6. However, as observed in *E. gracilis* Z (Zeng et al., 2016), *E. gracilis* 815 was found to exhibit a comparable cell density among all the conditions in result. Studies on *E. gracilis* Z under different culture conditions have shown that adding the carbon sources can promote biomass accumulation under dark conditions (Rodríguez-Zavala et al., 2006; Sun et al., 2018). In the present study, however, no significant difference was observed across conditions of *E. gracilis* 815 in biomass accumulation by Day 6, indicating that this new strain shows strong adaptability to environmental conditions when biomass is harvested in the short term. Since we have proved the possibility of water reuse in the cultivation of *E. gracilis* Z recently (Wu et al., 2021a), further evaluations in *E. gracilis* 815 could be expanded to reusing the wastes from other process, such as exhaust gases or wastewater from factories or livestock, in the cultivation of microalgae (Tsolcha et al., 2018; Amenorfenyo et al., 2019; Dębowski et al., 2020b; Geremia et al., 2021; Mathew et al., 2021; Cheng et al., 2022; Lopez-Sanchez et al., 2022; Singh and Singh, 2022).

The biomass of various *E. gracilis* 815 groups ranged between 0.78–0.94 g/L when their cell density ranged between 1.90 × 10^6^–2.3 × 10^6^ cells/ml. In comparison, the biomass of *E. gracilis* Z has been recorded to reach 6.61 g/L and 10.8 g/L at cell densities of 1.86 × 10^7^ cells/ml and 2.45 × 10^7^ cells/ml, respectively, with the initial inoculated densities at 1 × 10^6^ cells/ml. Due to the differences in initial inoculated densities and culture conditions between the experiment of *E. gracilis* 815 and the reference of *E. gracilis* Z (Zeng et al., 2016), modifications such as increasing the initial inoculated density or optimizing the cultivation conditions can be adopted to increase the biomass accumulation in *E. gracilis* 815, and are worthy of further research and exploration. When cultivated with the same initial inoculated densities at 1.1 × 10^6^ cells/ml, no significant difference of biomass was found between *E. gracilis* 815 and *E. gracilis* Z (Supplementary Figure S1). Previous studies have reported that glucose was more conducive to cell density and biomass accumulation than ethanol in *E. gracilis* Z (Afiukwa and Ogbonna, 2007; Kim et al., 2021). For example, the cell density of the mixotrophic group with ethanol was only half of that with glucose in *E. gracilis* Z after being cultivated for 6 days (Afiukwa and Ogbonna, 2007). However, there were no significant differences in cell density and biomass in *E. gracilis* 815. From the perspective of growth and biomass accumulation, we proposed that *E. gracilis* 815 has prospects for a wide range of applications and is a potential application-type algae strain.

Paramylon is a product of photosynthesis and the main product of carbon storage in *Euglena*. Previous work in *E. gracilis Z* reported that the highest paramylon content was about 200–250 mg/g on Day 4 under heterotrophic conditions (Zeng et al., 2016), which was close to the paramylon content of *E. gracilis* 815 in autotrophic conditions on Day 6. Garlaschi et al. (1974) have shown that ethanol can benefit the synthesis of paramylon in *E. gracilis Z*. The accumulation of paramylon was related to the consumption of ethanol in the medium (Garlaschi et al., 1974). Other studies identified that glucose was the best carbon source for the growth of *Chromochloris zofingiensis* and *E. gracilis* Z, owing to its ability to increase photosynthetic yield (Santek et al., 2010; Ivušić and Šantek, 2015; Roth et al., 2019). Here, by 6 Days of culture, the accumulation of *E. gracilis* 815 under light conditions was not significantly correlated with carbon source. Under dark heterotrophic conditions, the accumulation of paramylon in *E. gracilis* 815 using glucose was considerably higher than that achieved in light. Notably, Schwartzbach et al. (1975) showed that paramylon in *E. gracilis* Z and the non-photosynthetic mutant W3BUL induced by light, with darkness being conducive to paramylon synthesis (Schwartzbach et al., 1975). This agrees with the results of this paper. No significant differences were observed in the accumulation of paramylon when ethanol was added to the culture, suggesting that the accumulation of paramylon in *E. gracilis* 815 may not significantly depend on light or dark when using ethanol. However, as the biomass and total lipid accumulation among all the groups did not differ significantly, differences in paramylon accumulation may be related to differences in protein synthesis in *E. gracilis* 815*.*

Although there was no significant difference in total lipid accumulation across the non-autotrophic groups, total lipid contents ranged between 24.87–34.03%. Previous studies on other common lipid-producing algae yield have reported similar or even lower lipid contents. For instance, the typical lipid yield of *Chlorella* was 18.59–37.78%, the lipid content of *Scenedesmus obliquus* was 30.25–36.17%. In comparison, diatoms (*Nitzschia minor* and *Phaeodactylum tricornutum*) and *Isochrysis* were approximately 20% (Yang et al., 2011). Meanwhile, studies conducted in *Euglena* showed that the total lipid content of *E. gracilis* (NIES-48) varied within the range of 18–22% (Wang et al., 2018), and that of *Euglena* species varied within the scope of 10–30% (Mahapatra et al., 2013). Furthermore, Jung et al. (2021) found that the maximum lipid content of *E. gracilis* Z during autotrophic culture did not exceed 5%. Under mixotrophic and heterotrophic culture conditions, lipid accumulation in *E. gracilis* Z strain could not exceed 15 and 25%, respectively. Thus, *E. gracilis* 815 has a higher total lipids content than common oleaginous algae and *E. gracilis* Z (Jung et al., 2021). Moreover, *Euglena* species do not contain cell walls, which could significantly reduce the lipid production costs and enhance production efficiency.

Results showed that *E. gracilis* 815 accumulated significantly more total fatty acid content when using ethanol than either using glucose or photosynthetic, indicating that *E. gracilis* 815 has a higher utilization rate of ethanol in the fatty acid synthesis pathway. SAFAs of the ethanol mixotrophic group and two heterotrophic groups were significantly higher than the autotrophic group. In contrast, the autotrophic group and the glucose mixotrophic group promoted the production of PUFAs. According to the result, the addition of ethanol may be involved in the fatty acid saturation process of *E. gracilis* 815, and light may be involved in the fatty acid desaturation process of *E. gracilis* 815. Studies have also shown that light-induced culture can significantly increase the content of UFAs in *E. gracilis* Z, with SAFAs accounting for the primary product in the dark (Barsanti et al., 2000). This observation indicates that the synthesis of UFAs in *Euglena* is related to the photosynthetic activity.

Previous studies on *E. gracilis* Z have examined the contents of different types of fatty acids, and these results were compared with the levels achieved in *E. gracilis* 815. When *E. gracilis* Z was cultured to Day 7, SAFA contents under mixotrophic and heterotrophic conditions reached 23.7 and 88.3 mg/g dw, respectively (Zeng et al., 2016). In comparison, *E. gracilis* 815 obtained SAFA contents of 62.1 and 54.9 mg/g dw under mixotrophic and heterotrophic conditions on Day 6, respectively. *E. gracilis Z* has been reported to reach MUFA contents of up to 9.51 mg/g dw under mixotrophic conditions (Zeng et al., 2016), while *E. gracilis* 815 reached 17.8 mg/g dw when using ethanol in light. *E. gracilis Z* has reached PUFA contents of 94 and 42.6 mg/g dw in mixotrophic and heterotrophic conditions (Zeng et al., 2016), whereas *E. gracilis* 815 reached 50.2 mg/g dw in mixotrophic condition. Therefore, in comparison with *E. gracilis* Z, the novel strain *E. gracilis* 815 has a relatively higher capacity for producing MUFA, a relatively lower capacity for producing PUFA, and a similar power for making SAFA.

Since PUFAs have more functions than SAFAs, their value has received widespread attention. PUFAs are essential nutrients for the human body and will cause various diseases if they are lacking. PUFAs can thus be used as nutritional supplements in different food and beverages (Simopoulos, 2000; Nessel et al., 2020). In medicine, PUFAs serve a variety of physiological functions such as anti-inflammation and blood lipid regulation while also necessary for the prevention of cardiovascular diseases and treating schizophrenia (Marion-Letellier et al., 2015). PUFAs are also used in beauty and skin-care products. Here, various types of PUFAs were found in different non-autotrophic groups of *E. gracilis* 815, for instance α-linolenic acid, AA (arachidonic acid), EPA (eicosapentaenoic acid), and DHA (docosahexaenoic acid). As α-linolenic acid (C18:3n6) is a product of active photosynthesis (Reitz and Moore, 1972; Barsanti et al., 2000), it was higher in *E. gracilis* 815 cultured in light, regardless of carbon source. In comparison, the observed changes in the content of AA, EPA, and DHA do not seem to be directly related to the carbon source or light. Studies have shown that arachidonic acid and EPA synthesis do not depend on the light. The synthesis of these polyenoic acids is related to non-photosynthetic organelles, such as mitochondria and microsomes. When cultured with CM medium under mixotrophic conditions, *E. gracilis* Z contained 5.3–23.9, 3.9–5.8, and 0.7–3.4 mg/g dw of AA, EPA, and DHA, respectively, on Day 4 (Barsanti et al., 2000). Here, on Day 6 of *E. gracilis* 815 culture, the contents of these PUFAs were at 7.71–14.47, 1.53–3.79, and 0.8–2.4 mg/g dw, respectively. Another study culturing *E. gracilis* Z to the seventh day reported PUFA contents of 23.54, 6.07, and 5.95 mg/g dw in the mixotrophic condition, respectively. However, when *E. gracilis* 815 was cultured to the sixth day, the contents of these PUFAs were the highest in the mixotrophic group at 14.47, 3.79, and 2.4 mg/g dw, respectively (Zeng et al., 2016). While *E. gracilis* 815 produces slightly lower PUFAs than *E. gracilis* Z, the former may still be developed as an essential source of PUFA production. Overall, the differences in fatty acid composition between different carbon sources and trophic conditions in *E. gracilis* 815 were quite similar to *E. gracilis* Z, suggesting that these two strains shared similar a pattern of metabolic regulation (Reitz and Moore, 1972). Especially when environmental conditions are reasonably controlled and diversified metabolism characteristics are used, fatty acid production can be achieved purposefully for specific applications in *E. gracilis* 815.

In this study, some critical properties of biodiesels in *E. gracilis* 815 were also considered. Based on these indicators of feedstock oil, the actual production process can be assessed. Comparison of SN values of *E. gacilis* 815 to the American ASTM D6751 standard suggested that *E. gracilis* 815 is suitable for direct use as a biodiesel feedstock oil. The SN values of *E. gacilis* 815 were quite similar to those of other familiar sources of biodiesel, such as chicken fat (251.23), mutton fat (244.50), and waste fat oil (204.16) (Sakthivel et al., 2018). Only *E. gracilis* 815 using ethanol (in both light and dark conditions) and those using glucose in the dark met the standards required for IN and CN. If *E. gracilis* 815 were to be used as a biodiesel source under other conditions, problems such as diesel engine ignition difficulties and carbon deposits could emerge, making it unsuitable for direct use as feedstock oil. Notably, *E. gracilis* 815 using glucose in the dark reported the lowest IN and the highest CN. Both values reached the highest, most elevated international biodiesel standards and corresponded to reasonable levels of combustibility. Previous studies have conducted similar biodiesel evaluations on other industrial algae. For instance, *Haematococcus pluvialis*, a high commercial value algae strain, was evaluated under different environmental stresses and found to achieve SN values between 201.9–205.7, IN values between 100–120, and CN values between 45.9–51.6 (Lei et al., 2012). *Chlorella sorokiniana*, a potential biodiesel algae strain, was evaluated under different inoculation amounts and growth periods and found to achieve SN values between 197.8–199.4. The majority of IN values fell between 130–150, with the lowest at 113. CN values of only a few groups exceeded the minimum international standard of 45 (the highest CN value was 48.2) (Lu et al., 2012). Compared with these algae species, *E. gracilis* 815 has a superior CN value of 57.6 (when cultured on glucose in the dark). Totally, under most culture conditions, *E. gracilis* 815 attained SN, IN, and CN values that meet the required standards, suggesting a good biodiesel performance.

## Conclusion

In conclusion, a new strain of *E. gracilis*, *E. gracilis* 815, was found to have strong adaptability to the environment, fast-growing, relatively high biomass accumulation, and total lipid yield. The accumulation of biomass and total lipids showed no significant differences among all the groups. In contrast, the paramylon content in the mixotrophic group using glucose is lower than the heterotrophic group. Accumulation of total fatty acids and MUFAs in *E. gracilis* 815 was highest when cultured with ethanol in the presence of light. In contrast, when cultured in the dark, overall fatty acid production showed biodiesel potential. As a novel strain, further research is needed to ascertain the full biodiesel potential of *E. gracilis* 815. Future research on this strain could focus on combining transcriptomic and metabolic analyses to explore the mechanisms, determine the best environmental conditions for accumulating active substances, and achieve the production goals in a rapid, controllable, purposeful, and large-scale manner. Overall, *E. gracilis* 815 has demonstrated the potential of industrial production capacity and prospects for broad applications, making it an ideal candidate for industrial biodiesel.

## Data Availability

The original contributions presented in the study are included in the article/Supplementary Material, further inquiries can be directed to the corresponding author.

## References

[B1] Afiukwa C. A. Ogbonna J. C. (2007). Effects of Mixed Substrates on Growth and Vitamin Production by *Euglena Gracilis* . Afr. J. Biotechnol. 6 (22), 2612–2615. 10.5897/AJB2007.000-2417

[B2] Amenorfenyo D. K. Huang X. Zhang Y. Zeng Q. Zhang N. Ren J. (2019). Microalgae Brewery Wastewater Treatment: Potentials, Benefits and the Challenges. Ijerph 16 (11), 1910. 10.3390/ijerph16111910 PMC6603649 31151156

[B3] Barsanti L. Bastianini A. Passarelli V. Tredici M. R. Gualtieri P. (2000). Fatty Acid Content in Wild Type and WZSL Mutant of *Euglena Gracilis* - Effects of Carbon Source and Growth Conditions. J. Appl. Phycology 12 (3-5), 515–520. 10.1023/a:1008187514624

[B4] Bligh E. G. Dyer W. J. (1959). A Rapid Method of Total Lipid Extraction and Purification. Can. J. Biochem. Physiol. 37 (8), 911–917. 10.1139/o59-099 13671378

[B5] Brennan L. Owende P. (2010). Biofuels from Microalgae-A Review of Technologies for Production, Processing, and Extractions of Biofuels and Co-products. Renew. Sustainable Energ. Rev. 14 (2), 557–577. 10.1016/j.rser.2009.10.009

[B6] Chandra R. Iqbal H. M. N. Vishal G. Lee H.-S. Nagra S. (2019). Algal Biorefinery: A Sustainable Approach to Valorize Algal-Based Biomass towards Multiple Product Recovery. Bioresour. Technology 278, 346–359. 10.1016/j.biortech.2019.01.104 30718075

[B7] Cheng P. Li Y. Wang C. Guo J. Zhou C. Zhang R. (2022). Integrated marine Microalgae Biorefineries for Improved Bioactive Compounds: A Review. Sci. Total Environ. 817, 152895. 10.1016/j.scitotenv.2021.152895 34998757

[B8] Chew K. W. Yap J. Y. Show P. L. Suan N. H. Juan J. C. Ling T. C. (2017). Microalgae Biorefinery: High Value Products Perspectives. Bioresour. Technology 229, 53–62. 10.1016/j.biortech.2017.01.006 28107722

[B9] Chisti Y. (2007). Biodiesel from Microalgae. Biotechnol. Adv. 25 (3), 294–306. 10.1016/j.biotechadv.2007.02.001 17350212

[B10] Coleman L. W. Rosen B. H. Schwartzbach S. D. (1988). Environmental Control of Carbohydrate and Lipid Synthesis in *Euglena* . Plant Cell Physiol. 29, 423–432. 10.1093/oxfordjournals.pcp.a077510

[B11] Cramer M. Myers J. (1952). Growth and Photosynthetic Characteristics of *Euglena Gracilis* . Archiv Für Mikrobiologie 17 (1-4), 384–402. 10.1007/bf00410835

[B12] de Carvalho Silvello M. A. Severo Gonçalves I. Patrícia Held Azambuja S. Silva Costa S. Garcia Pereira Silva P. Oliveira Santos L. (2022). Microalgae-based Carbohydrates: A green Innovative Source of Bioenergy. Bioresour. Technology 344 (Pt B), 126304. 10.1016/j.biortech.2021.126304 34752879

[B13] Dębowski M. Zieliński M. Kazimierowicz J. Kujawska N. Talbierz S. (2020a). Microalgae Cultivation Technologies as an Opportunity for Bioenergetic System Development—Advantages and Limitations. Sustainability 12 (23), 9980. 10.3390/su12239980

[B14] Dębowski M. Zieliński M. Kisielewska M. Kazimierowicz J. Dudek M. Świca I. (2020b). The Cultivation of Lipid-Rich Microalgae Biomass as Anaerobic Digestate Valorization Technology—A Pilot-Scale Study. Processes 8 (5), 517. 10.3390/pr8050517

[B15] Garlaschi F. M. Garlaschi A. M. Lombardi A. Forti G. (1974). Effect of Ethanol on the Metabolism of *Euglena Gracilis* . Plant Sci. Lett. 2 (1), 29–39. 10.1016/0304-4211(74)90035-2

[B16] Geremia E. Ripa M. Catone C. M. Ulgiati S. (2021). A Review about Microalgae Wastewater Treatment for Bioremediation and Biomass Production-A New Challenge for Europe. Environments 8 (12), 136. 10.3390/environments8120136

[B17] Gupta S. P. KhushbooGupta V. K. Gupta V. K. Minhas U. Kumar R. Sharma B. (2021). *Euglena* Species: Bioactive Compounds and Their Varied Applications. Ctmc 21 (29), 2620–2633. 10.2174/1568026621666210813111424 34392825

[B18] Harwood J. L. Guschina I. A. (2009). The Versatility of Algae and Their Lipid Metabolism. Biochimie 91 (6), 679–684. 10.1016/j.biochi.2008.11.004 19063932

[B19] Hurlbert R. E. Rittenberg S. C. (1962). Glucose Metabolism of *Euglena gracilisvar*. Bacillaris; Growth and Enzymatic Studies*. J. Protozool 9, 170–182. 10.1111/j.1550-7408.1962.tb02602.x 14450133

[B20] Ivušić F. Šantek B. (2015). Optimization of Complex Medium Composition for Heterotrophic Cultivation of *Euglena Gracilis* and Paramylon Production. Bioproc. Biosyst Eng 38 (6), 1103–1112. 10.1007/s00449-015-1353-3 25601569

[B21] Jung J.-M. Kim J. Y. Jung S. Choi Y.-E. Kwon E. E. (2021). Quantitative Study on Lipid Productivity of *Euglena Gracilis* and its Biodiesel Production According to the Cultivation Conditions. J. Clean. Prod. 291, 125218. 10.1016/j.jclepro.2020.125218

[B22] Kang N. K. Baek K. Koh H. G. Atkinson C. A. Ort D. R. Jin Y.-S. (2022). Microalgal Metabolic Engineering Strategies for the Production of Fuels and Chemicals. Bioresour. Technology 345, 126529. 10.1016/j.biortech.2021.126529 34896527

[B23] Kim S. Wirasnita R. Lee D. Yu J. Lee T. (2021). Enhancement of Growth and Paramylon Production of *Euglena Gracilis* by Upcycling of Spent Tomato Byproduct as an Alternative Medium. Appl. Sci. 11 (17), 8182. 10.3390/app11178182

[B66] Koonin S. E. (2006). Getting Serious About Biofuels. Science 311 (5760), 435. 10.1126/science.1124886 16439624

[B24] Krisnangkura K. (1986). A Simple Method for Estimation of Cetane index of Vegetable Oil Methyl Esters. J. Am. Oil Chem. Soc. 63 (4), 552–553. 10.1007/bf02645752

[B25] Lam M. K. Lee K. T. (2012). Microalgae Biofuels: A Critical Review of Issues, Problems and the Way Forward. Biotechnol. Adv. 30 (3), 673–690. 10.1016/j.biotechadv.2011.11.008 22166620

[B26] Lei A. Chen H. Shen G. Hu Z. Chen L. Wang J. (2012). Expression of Fatty Acid Synthesis Genes and Fatty Acid Accumulation in *Haematococcus pluvialis* under Different Stressors. Biotechnol. Biofuels 5 (1), 18. 10.1186/1754-6834-5-18 22448811 PMC3337298

[B65] Li Y. Horsman M. Wu N. Lan C. Q. Dubois-Calero N. (2008). Biofuels From Microalgae. Biotechnol. Prog. 24 (4), 815–820. 10.1021/bp070371k 18335954

[B27] Li S. Li X. Ho S.-H. (2022). Microalgae as a Solution of Third World Energy Crisis for Biofuels Production from Wastewater toward Carbon Neutrality: An Updated Review. Chemosphere 291 (Pt 1), 132863. 10.1016/j.chemosphere.2021.132863 34774903

[B28] López-Sánchez A. Silva-Gálvez A. L. Aguilar-Juárez Ó. Senés-Guerrero C. Orozco-Nunnelly D. A. Carrillo-Nieves D. (2022). Microalgae-based Livestock Wastewater Treatment (MbWT) as a Circular Bioeconomy Approach: Enhancement of Biomass Productivity, Pollutant Removal and High-Value Compound Production. J. Environ. Manage. 308, 114612. 10.1016/j.jenvman.2022.114612 35149401

[B29] Lu S. Wang J. Niu Y. Yang J. Zhou J. Yuan Y. (2012). Metabolic Profiling Reveals Growth Related FAME Productivity and Quality of *Chlorella Sorokiniana* with Different Inoculum Sizes. Biotechnol. Bioeng. 109 (7), 1651–1662. 10.1002/bit.24447 22252441

[B30] Luo W. Yuan Z. H. Liao C. P. (2006). Biodiesel Standard and Quality Assessment. Yingkou, China: Renewable Energy 4 , 33–37.

[B31] Mahapatra D. M. Chanakya H. N. Ramachandra T. V. (2013). *Euglena* Sp. As a Suitable Source of Lipids for Potential Use as Biofuel and Sustainable Wastewater Treatment. J. Appl. Phycol 25 (3), 855–865. 10.1007/s10811-013-9979-5

[B32] Marion-Letellier R. Savoye G. Ghosh S. (2015). Polyunsaturated Fatty Acids and Inflammation. Iubmb Life 67 (9), 659–667. 10.1002/iub.1428 26397837

[B33] Mathew M. M. Khatana K. Vats V. Dhanker R. Kumar R. Dahms H.-U. (2021). Biological Approaches Integrating Algae and Bacteria for the Degradation of Wastewater Contaminants-A Review. Front. Microbiol. 12, 801051. 10.3389/fmicb.2021.801051 35185825 PMC8850834

[B34] Mohibbeazam M. Waris A. Nahar N. (2005). Prospects and Potential of Fatty Acid Methyl Esters of Some Non-traditional Seed Oils for Use as Biodiesel in India. Biomass and Bioenergy 29 (4), 293–302. 10.1016/j.biombioe.2005.05.001

[B35] Nessel I. De Rooy L. Khashu M. Murphy J. L. Dyall S. C. (2020). Long‐Chain Polyunsaturated Fatty Acids and Lipid Peroxidation Products in Donor Human Milk in the United Kingdom: Results from the LIMIT 2‐Centre Cross‐Sectional Study. J. Parenter. Enteral Nutr. 44 (8), 1501–1509. 10.1002/jpen.1773 32048312

[B36] Ramos M. J. Fernández C. M. Casas A. Rodríguez L. Pérez Á. (2009). Influence of Fatty Acid Composition of Raw Materials on Biodiesel Properties. Bioresour. Technology 100 (1), 261–268. 10.1016/j.biortech.2008.06.039 18693011

[B37] Reitz R. C. Moore G. S. (1972). Effects of Changes in the Major Carbon Source on the Fatty Acids of *Euglena gracilis* . Lipids 7 (3), 217–220. 10.1007/BF02533068 4623226

[B38] Rodríguez-Zavala J. S. Ortiz-Cruz M. A. Mendoza-Hernández G. Moreno-Sánchez R. (2010). Increased Synthesis of α-tocopherol, Paramylon and Tyrosine by *Euglena Gracilis* under Conditions of High Biomass Production. J. Appl. Microbiol. 109 (6), 2160–2172. 10.1111/j.1365-2672.2010.04848.x 20854454

[B39] Rodríguez-Zavala J. S. Ortiz-Cruz M. A. Moreno-Sanchez R. (2006). Characterization of an Aldehyde Dehydrogenase from *Euglena Gracilis* . J. Eukaryot. Microbiol. 53 (1), 36–42. 10.1111/j.1550-7408.2005.00070.x 16441583

[B40] Roth M. S. Westcott D. J. Iwai M. Niyogi K. K. (2019). Hexokinase Is Necessary for Glucose-Mediated Photosynthesis Repression and Lipid Accumulation in a green Alga. Commun. Biol. 2, 347. 10.1038/s42003-019-0577-1 31552300 PMC6753101

[B41] Santek B. Felski M. Friehs K. Lotz M. Flaschel E. (2010). Production of Paramylon, a β-1,3-glucan, by Heterotrophic Cultivation of *Euglena gracilis* on Potato Liquor. Eng. Life Sci. 10 (2), NA. 10.1002/elsc.200900077

[B42] Sakthivel R. Ramesh K. Purnachandran R. Mohamed Shameer P. (2018). A Review on the Properties, Performance and Emission Aspects of the Third Generation Biodiesels. Renew. Sustainable Energ. Rev. 82, 2970–2992. 10.1016/j.rser.2017.10.037

[B43] Schwartzbach S. D. Schiff J. A. Goldstein N. H. (1975). Events Surrounding the Early Development of *Euglena* Chloroplasts. Plant Physiol. 56 (2), 313–317. 10.1104/pp.56.2.313 16659293 PMC541810

[B44] Shao Q. Hu L. Qin H. Liu Y. Tang X. Lei A. (2019). Metabolomic Response of *Euglena Gracilis* and its Bleached Mutant Strain to Light. PLoS One 14 (11), e0224926. 10.1371/journal.pone.0224926 31697795 PMC6837420

[B45] Simopoulos A. P. (2000). Human Requirement for N-3 Polyunsaturated Fatty Acids. Poult. Sci. 79 (7), 961–970. 10.1093/ps/79.7.961 10901194

[B46] Singh A. Singh A. (2022). Microbial Degradation and Value Addition to Food and Agriculture Waste. Curr. Microbiol. 79 (4), 119. 10.1007/s00284-022-02809-5 35235053

[B47] Sun A. Hasan M. T. Hobba G. Nevalainen H. Te′o J. (2018). Comparative Assessment of the *Euglena Gracilis* Var. Saccharophila Variant Strain as a Producer of the β‐1,3‐glucan Paramylon under Varying Light Conditions. J. Phycol. 54 (4), 529–538. 10.1111/jpy.12758 29889303

[B48] Suzuki K. Mitra S. Iwata O. Ishikawa T. Kato S. Yamada K. (2015). Selection and Characterization of *Euglena Anabaena* Var. Minor as a New Candidate *Euglena* Species for Industrial Application. Biosci. Biotechnol. Biochem. 79 (10), 1730–1736. 10.1080/09168451.2015.1045828 25988946

[B49] Takenaka S. Kondo T. Nazeri S. Tamura Y. Tokunaga M. Tsuyama S. (1997). Accumulation of Trehalose as a Compatible Solute under Osmotic Stress in *Euglena Gracilis* Z. J. Eukaryot. Microbiol. (Usa) 44, 609–613. 10.1111/j.1550-7408.1997.tb05967.x

[B50] Thuillier-Bruston F. Briand J. Laval-Martin D. (1990). Effects of a First Exposure to Ethanol on the Compositions of Neutral and Polar Lipids in *Euglena Gracilis* Z, Taken as a Hepatic Cell Model: Equilibration by Citrulline-Malate. Biochem. Med. Metab. Biol. 44 (2), 159–174. 10.1016/0885-4505(90)90057-8 2252617

[B51] Tsolcha O. Tekerlekopoulou A. Akratos C. Aggelis G. Genitsaris S. Moustaka-Gouni M. (2018). Agroindustrial Wastewater Treatment with Simultaneous Biodiesel Production in Attached Growth Systems Using a Mixed Microbial Culture. Water 10 (11), 1693. 10.3390/w10111693

[B52] Vicente G. Martínez M. Aracil J. (2007). Optimisation of Integrated Biodiesel Production. Part I. A Study of the Biodiesel Purity and Yield. Bioresour. Technology 98 (9), 1724–1733. 10.1016/j.biortech.2006.07.024 16934452

[B53] Wang J. Chen L. Tian X. Gao L. Niu X. Shi M. (2013). Global Metabolomic and Network Analysis of *Escherichia coli* Responses to Exogenous Biofuels. J. Proteome Res. 12 (11), 5302–5312. 10.1021/pr400640u 24016299

[B54] Wang Y. Seppänen-Laakso T. Rischer H. Wiebe M. G. (2018). *Euglena Gracilis* Growth and Cell Composition under Different Temperature, Light and Trophic Conditions. PLoS One 13 (4). e0195329. 10.1371/journal.pone.0195329 29649233 PMC5896972

[B64] Williams P. J. (2007). Biofuel: microalgae cut the social and ecological costs. Nature 450 (7169). 478. 10.1038/450478a 18033275

[B55] Wu M. Du M. Wu G. Lu F. Li J. Lei A. (2021a). Water Reuse and Growth Inhibition Mechanisms for Cultivation of Microalga *Euglena Gracilis* . Biotechnol. Biofuels 14 (1), 132. 10.1186/s13068-021-01980-4 34090512 PMC8180174

[B56] Wu M. Li J. Qin H. Lei A. Zhu H. Hu Z. (2020). Pre-concentration of Microalga *Euglena Gracilis* by Alkalescent pH Treatment and Flocculation Mechanism of Ca_3_(PO4)_2_, Mg_3_(PO4)_2_, and Derivatives. Biotechnol. Biofuels 13 (3), 98. 10.1186/s13068-020-01734-8 32514310 PMC7260821

[B57] Wu M. Qin H. Deng J. Liu Y. Lei A. Zhu H. (2021b). A New Pilot-Scale Fermentation Mode Enhances *Euglena Gracilis* Biomass and Paramylon (β-1,3-Glucan) Production. J. Clean. Prod. 321, 128996. 10.1016/j.jclepro.2021.128996

[B58] Wu X. Ruan R. Du Z. Liu Y. (2012). Current Status and Prospects of Biodiesel Production from Microalgae. Energies 5 (8), 2667–2682.

[B59] Yang J. Jiang J. C. Zhang N. (2011). Oil Productivity Capabilities of Several Microalgae Strains in Different Cultivation Methods. Nanjing, China: Biomass Chemical Engineering 45 (02), 15–19.

[B60] Zakryś B. Milanowski R. Karnkowska A. (2017). Evolutionary Origin of *Euglena* . Adv. Exp. Med. Biol. 979, 3–17. 10.1007/978-3-319-54910-1_1 28429314

[B61] Zeng M. Hao W. Zou Y. Shi M. Jiang Y. Xiao P. (2016). Fatty Acid and Metabolomic Profiling Approaches Differentiate Heterotrophic and Mixotrophic Culture Conditions in a Microalgal Food Supplement ‘*Euglena*’. BMC Biotechnol. 16 (1), 49. 10.1186/s12896-016-0279-4 27255274 PMC4890288

[B62] Zimorski V. Rauch C. van Hellemond J. J. Tielens A. G. M. Martin W. F. (2017). The Mitochondrion of *Euglena Gracilis* . Adv. Exp. Med. Biol. 979, 19–37. 10.1007/978-3-319-54910-1_2 28429315

