# Supplementary figures and images for: Evaluation of Euglena gracilis 815 as a New Candidate for Biodiesel Production
Source: Front Bioeng Biotechnol. 2022 Mar 25;10:827513. doi: 10.3389/fbioe.2022.827513 (PMC8990129; doi:10.3389/fbioe.2022.827513)

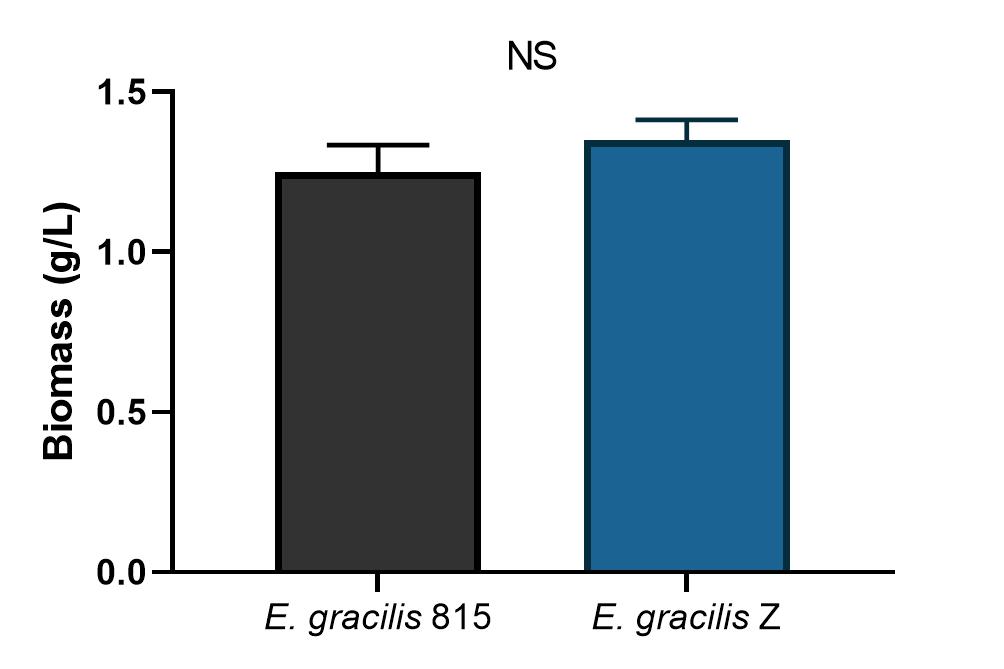

Supplement: Supplementary file 1 [file Image1.JPEG]
